# Erythrocyte n-6 Fatty Acids and Risk for Cardiovascular Outcomes and Total Mortality in the Framingham Heart Study

**DOI:** 10.3390/nu10122012

**Published:** 2018-12-19

**Authors:** William S. Harris, Nathan L. Tintle, Vasan S. Ramachandran

**Affiliations:** 1 Department of Internal Medicine, Sanford School of Medicine, University of South Dakota, Vermillion, SD 57069, USA; 2 OmegaQuant Analytics, LLC, Sioux Falls, SD 57106, USA; 3 Department of Mathematics & Statistics, Dordt College, Sioux Center, IA 51250, USA; nathan.tintle@dordt.edu; 4 National Heart Lung and Blood Institute’s and Boston University’s Framingham Heart Study, Framingham, MA 02118, USA; vasan@bu.edu; 5 Departments of Cardiology and Preventive Medicine, Department of Medicine, Boston University School of Medicine, Boston, MA 02118, USA; 6 Department of Biostatistics, Boston University School of Public Health, Boston, MA 02118, USA

**Keywords:** epidemiology, prospective cohort study, n-6 fatty acids, n-3 fatty acids, linoleic acid, arachidonic acid

## Abstract

Background: The prognostic value of erythrocyte levels of n-6 fatty acids (FAs) for total mortality and cardiovascular disease (CVD) outcomes remains an open question. Methods: We examined cardiovascular (CV) outcomes and death in 2500 individuals in the Framingham Heart Study Offspring cohort without prevalent CVD (mean age 66 years, 57% women) as a function of baseline levels of different length n-6 FAs (18 carbon, 20 carbon, and 22 carbon) in the erythrocyte membranes. Clinical outcomes were monitored for up to 9.5 years (median follow up, 7.26 years). Cox proportional hazards models were adjusted for a variety of demographic characteristics, clinical status, and red blood cell (RBC) n-6 and long chain n-3 FA content. Results: There were 245 CV events, 119 coronary heart disease (CHD) events, 105 ischemic strokes, 58 CVD deaths, and 350 deaths from all causes. Few associations between either mortality or CVD outcomes were observed for n-6 FAs, with those that were observed becoming non-significant after adjusting for n-3 FA levels. Conclusions: Higher circulating levels of marine n-3 FA levels are associated with reduced risk for incident CVD and ischemic stroke and for death from CHD and all-causes; however, in the same sample little evidence exists for association with n-6 FAs. Further work is needed to identify a full profile of FAs associated with cardiovascular risk and mortality.

## 1. Introduction

The roles of n-3 and/or n-6 polyunsaturated fatty acids (PUFAs) in health and disease are controversial. Whereas most studies have reported that higher circulating levels of the long-chain n-3 PUFAs (i.e., eicosapentaenoic acid (EPA) and docosahexaenoic acid (DHA)) are protective against cardiovascular disease (CVD) and premature death [1], the story is not as clear for the n-6 PUFAs. Previous studies have reported that higher intakes of linoleic acid (LA) and higher circulating levels of arachidonic acid (AA) are associated with cardiovascular (CV) benefit [1,2]. In addition, LA blood levels are inversely associated with risk for type 2 diabetes mellitus [3]. Nevertheless, some warn that current intakes (and thus blood levels) of the n-6 PUFAs, especially these two major fatty acids (FAs) in the n-6 family, are dangerous [4,5]. The proposed mechanism of harm is via increased inflammation, driven by the conversion of AA into pro-inflammatory/pro-thrombotic eicosanoids such as prostaglandin E2, thromboxane A2, and leukotriene B4 [6]. Accordingly, ratios like n-6:n-3 [7] or AA:EPA [8] have been promoted as better summary metrics of PUFA status than either family alone. This perspective has, however, been criticized on both theoretical [9,10,11,12] and evidential [13,14,15] bases.

In a previous report from the Framingham Offspring cohort, we examined relations between red blood cell (RBC) n-3 FAs, specifically EPA and DHA (the sum of which is called the omega-3 index [16]) and risk for CVD and total mortality [17]. In this follow-up analyses, we focus on the n-6 FA family and their relations with these outcomes.

## 2. Materials and Methods

### 2.1. Methods

The Framingham Heart Study is a longitudinal community-based cohort study that was initiated in 1948. The selection criteria for the Framingham Offspring Cohort and the Framingham Omni Cohort have previously been described [18,19]. Briefly, adult children of the original cohort were recruited in 1971 into the Framingham Offspring Cohort. We evaluated Framingham Offspring participants (*n =* 3021) who attended their eighth examination cycle (2005–2008). Participants were excluded in hierarchical order if they were missing RBC fatty acid measurements or clinical covariates (*n* = 122), or having prevalent cardiovascular disease (e.g., nonfatal coronary heart disease (CHD) or stroke; *n* = 350), leaving *n* = 2500 for this analysis. The study protocol was approved by the Institutional Review Board of the Boston University Medical Center. Informed consent was provided by all participants.

### 2.2. Covariates and Mortality Outcomes

We considered seventeen primary baseline demographic and cardiovascular risk covariates: sex, age, body mass index (BMI), marital status, education level, employment status, health insurance status, regular aspirin use, prevalent hypertensive status, use of cholesterol medication, prevalent diabetes, alcohol consumption, smoking status, metabolic equivalent (METS), total to high-density lipoprotein (HDL) cholesterol ratio, systolic blood pressure and C-reactive protein. Seven endpoints were examined: four mortality-related outcomes ((1) any mortality, (2) CVD mortality (CHD death or sudden cardiac death), (3) cancer mortality, or (4) death from non-CVD or cancer causes) and three cardio-vascular related outcomes ((1) total stroke (any fatal or non-fatal ischemic stroke), (2) total CHD (fatal or non-fatal myocardial infarction (MI), or CHD death or sudden cardiac death), and (3) total CVD (any stroke, CHD or CHD mortality)). We focused our analyses on seven RBC FAs from the n-6 family: 18:2n6 (linoleic acid, LA), 18:3n6 (gamma-linolenic acid, GLA), 20:2n6 (eicosadienoic acid, EDA), 20:3n6 (dihomo-gamma linolenic acid, DGLA), 20:4n6 (arachidonic acid, AA), 22:4n6 (adrenic acid, ADA) and 22:5n6 (docosapentaenoic acid, DPA n-6). In some analyses, we adjusted for the omega-3 index (EPA + DHA).

### 2.3. RBC Fatty Acid Analysis

Blood was drawn after a 10–12 h fast into an EDTA tube, and RBCs were separated from plasma by centrifugation. The RBC fraction was frozen at −80 °C immediately after collection. RBC fatty acid composition was determined as described previously [20]. Briefly, RBCs were incubated at 100 °C with boron trifluoride-methanol and hexane to generate fatty acid methyl esters that were then analyzed by gas chromatography with flame ionization detection. The coefficients of variation for the seven n-6 FAs measured were <3% for 18:2n6, 20:3n6, 20:4n6, and 22:4n6; 7% for 22:5n6; 11% for 20:2n6 and 21% for 18:3n6.

### 2.4. Statistical Analysis

Description of sample characteristics was conducted using standard statistical metrics (e.g., means, SDs, correlations). Hazard ratios were estimated using the survival package in R [21]. Primary analyses predicted incident clinical outcomes (date of event (CVD or death) or date of censoring) by groups of n-6 FAs (the 18-carbon, 20-carbon, and 22-carbon moities), with follow-up analyses adjusting for a variety of demographic covariates, and finally for the omega-3 index. Secondary analyses explored the relationship between event risk and n-6 FA levels by quintiles, individual omega-6 FA levels, and the sum of all omega-6 FAs. All analyses used two-sided tests at the 0.05 significance level.

## 3. Results

### 3.1. Cohort Description

The Framingham Offspring study consists of 2500 individuals for whom FAs, clinical outcomes, and demographic covariates were available, and who also did not have prevalent CVD at baseline. At baseline, the average age was 66 and the sample contained slightly more females (57%) than males (43%). The sample was fairly well educated (over two-thirds of the sample had at least some college education), with approximately half of the sample employed and the majority of the remainder having retired. Most (88%) of the sample had insurance at baseline and the distribution of cardiometabolic traits followed expected distributions, with approximately 40% of the sample using aspirin regularly, with similar numbers with prevalent hypertension, and high cholesterol. Rates of prevalent diabetes was lower (13%). The majority of the sample periodically consumed alcohol, and the vast majority (over 90%) were nonsmokers. Table 1 provides a complete overview of the demographic profile of the sample. Supplemental Table S1 illustrates the correlation between and among the major n-3 and n-6 fatty acids in this sample. While correlations are generally higher within the n-3 or n-6 fatty acids than between, this is not always the case (e.g., moderate negative correlations between both 20:2n6, 20:3n6, and 20:4n6 levels). Supplemental Table S2 illustrates the number of events present in the sample with 350 of the participants having died from any cause during the course of the study, but only 58 of those deaths were attributable to CVD. The maximum time to follow-up was approximately 9 years.

### 3.2. Omega-6 Fatty Acids and Mortality and Cardiovascular Disease Risk

**Figure 1:**
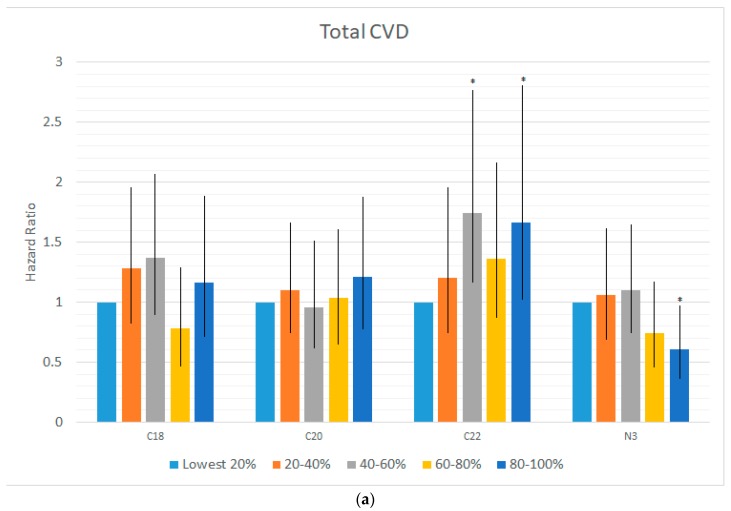

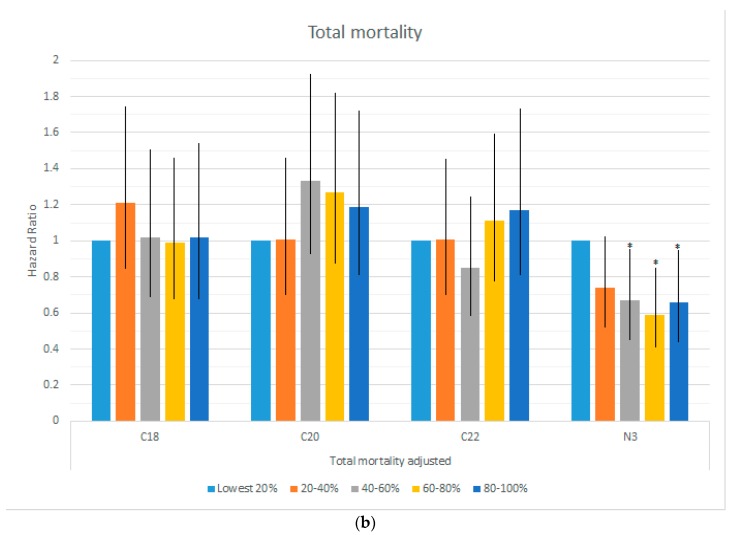
(**a**) Hazard ratios for total cardiovascular (CVD) risk by n-6 FAs and the omega-3 index. Figure 1a illustrates the associations between n-6 or n-3 fatty acids and total CVD risk. Bar heights are hazard ratios with 95% confidence intervals provided for each hazard ratio. Hazard ratios are provided for each quintile are all relative to the risk within the lowest quintile. Statistically significant HRs at the 0.05 level are denoted with asterisks. Hazard ratios depicted in this figure are from models adjusting for all demographic variables in Table 1, but do not adjust for the omega-3 index and/or other omega-6 FAs. There is no significant linear trend for the 18-carbon n-6 FAs (*p* = 0.68) or the 20-carbon n-6 FAs (*p* = 0.52), while the 22-carbon n-6 FAs (*p* = 0.031) and the omega-3 index (*p* = 0.008) both have significant linear trends across the 5 quintile groups (see Table 2a–c for additional details). Finally, we note that, as shown in Table 2c, both linear associations as well as all individual quintile group associations become non-significant (*p* > 0.05) for both the omega-3 index and 22 carbon n-6 FAs when both are simultaneously entered into the statistical model. (**b**) Hazard ratios for total mortality (CVD) risk by n-6 FAs and the omega-3 index. Figure 1b illustrates the associations between n-6 or n-3 fatty acids and Total mortality. Bar heights are hazard ratios with 95% confidence intervals provided for each hazard ratio. Hazard ratios are provided for each quintile are all relative to the risk within the lowest quintile. Statistically significant HRs at the 0.05 level are denoted with asterisks. Hazard ratios depicted in this figure are from models adjusting for all demographic variables in Table 1, but do not adjust for the omega-3 index and/or other omega-6 FAs. There is no significant linear trend for the 18 carbon n-6 FAs (*p* = 0.69), the 20 carbon n-6 FAs (*p* = 0.16) and the 22 carbon n-6 FAs (*p* = 0.32), while, the omega-3 index (*p* = 0.02) has a significant linear trend across the 5 quintile groups. Finally, we note that, as shown in Table 2c, linear associations as well as all individual quintile group associations become non-significant (*p* > 0.05) for all n-6 fatty acids when adjusting for the omega-3 index, while the omega-3 index remains significant (*p* < 0.05) in all models.

Across all three classes of n-6 FAs (18-carbon, 20-carbon and 22-carbon) little evidence of association with cardiovascular outcomes or mortality was observed. In unadjusted analyses, the 18-carbon n-6′s showed an association with reduced risk of CVD and other (non-CVD, non-cancer) mortality, however, these associations became non-significant after adjusting for demographic variables (Table 2a). No associations were detected before or after evaluating 20-carbon n-6s (Table 2b). While 22-carbon n-6′s showed some evidence of positive association with Total CVD, CHD, and stroke risk after adjusting for demographic covariates, all of these associations became non-significant after also adjusting for the omega-3 index (Table 2c). Notably, the omega-3 index remained significant in many of the models for CVD event risk and CVD, non-cancer/non-CVD and all-cause mortality (Table 2a–c; Figure 1).

### 3.3. Additional Fatty Acid Analyses

Supplemental Table S3a–g illustrate parallel analyses for each of the seven individual n-6 FAs. In short, these analyses follow what was observed for the classes of FAs. Namely, few associations were observed between the individual FAs and either cardiovascular events or mortality and what few associations were observed in unadjusted models tended to become non-significant in models that adjusted for demographic covariates. One exception was the positive association between ESA (C20:2n6) and all-cause mortality, a relationship which remained even after adjusting for the omega-3 index (Table S3c). Other exceptions were the positive associations between DTA (C22:4n6) and stroke, and the positive associations between DPA (C22:5n6) and all-cause mortality and non-CVD/non-cancer mortality, though these associations all became non-significant after adjusting for the omega-3 index (Table S3f–g). An analysis on the sum of all seven omega-6 FAs identified no significant associations after adjusting for the omega-3 index.

## 4. Discussion

In this study of participants in the Framingham Offspring study, the n-6 PUFAs were generally unrelated with vital status, especially in fully adjusted models accounting for subject characteristics and the omega-3 index. The only exception was for EDA which was directly related with risk all-cause mortality. The omega-3 index was inversely associated with most of the outcomes examined here even after adjustment for the n-6 FAs, whether in the full aggregate, the carbon-chain groups, or the individual n-6 FAs.

Delgado et al. recently reported the relations between red blood cell (RBC) n-6 FA levels and risk for death from all causes [22]. Using data from 3259 patients undergoing diagnostic coronary artery catheterization and followed for 10 years, these investigators found that RBC levels of n-6 FAs were inversely related with risk for death. However, a more granular examination revealed widely varying associations with n-6 FAs depending on chain length. The 18-carbon species (LA and GLA) were inversely associated with death, those with 20 carbons (AA and DGLA) were largely unrelated with risk, and those with 22 carbons (ADA and DPAn-6) were directly related with risk. A case-control study using RBC FA profiles from acute coronary syndrome patients found that a suite of 10 RBC FAs was able to predict case status better than the classical risk factors included in the Framingham Risk Score [23]. In that study, LA, AA, and GLA were predictors of lower risk for coronary disease, whereas EDA was associated with higher risk. In yet another study, RBC both EPA and DPAn-6 added independent predictive power to a standard algorithm used to predict risk for death in post myocardial infarction patients [24]. All of these studies suggest that n-6 FAs are not all created equal, and that to speak of them as a monolithic class of FAs with similar biochemical functions and physiological roles is probably incorrect. In this study, we further illustrated a widely varying correlation structure between n-3 FAs and n-6 FAs, as well as within the n-6 FAs, further supporting the argument that not each n-6 FA may have its own unique role to play in metabolism. However, one important trend worth noting is that, for 22 carbon FAs, associations with cardiovascular outcomes which were significant even after adjusting for demographic variables, became non-significant after adjusting for n-3 FAs. In these models (Table2c, fully adjusted model), n-3 FAs were significant, suggesting that the initial n-6 associations with cardiovascular outcomes were better explained by variation in n-3 FAs. Further work is needed to better understand the role of the full profile of fatty acids on disease risk.

The EDA finding in the present study was somewhat unexpected. EDA levels were adversely associated risk for acute coronary syndromes in a case-control study from Kansas City [23]. On the other hand, serum EDA levels were recently reported to be reduced in patients with inflammatory bowel disease [25], and in a large prospective study of plasma phospholipid FAs and risk for type 2 diabetes, EDA was inversely associated with incident disease [26]. These findings—along with those of the present study—would suggest that EDA participates in some manner in pathophysiology of these conditions. EDA is an elongation product of LA (C18 to C20, both with 2 double bonds), and on the biosynthetic path to DGLA and AA. These latter two n-6 FAs serve as substrates for the production of a wide variety of active metabolites called oxylipins [27], but whether the beneficial/harmful effects of EDA (if they indeed exist) are mediated by oxylipin metabolism is unknown. Complicating these inconsistent observations is the fact that levels of EDA in these subjects were very low, averaging only about 0.25% of total RBC FAs. Consequently, the ultimate meaning and impact of EDA in biology is unclear.

### Strengths and Limitations

Strengths of this study include the large sample size and number of events, the unambiguous nature of the primary endpoint, the inclusion of community dwellers (instead of a patient population), and the use of an objective biomarker of PUFA exposure with low biological variability [28]. The previous detection of clear inverse relations between the omega-3 index and outcomes in this same cohort suggests that our failure to see associations with the n-6 FAs was not due to a lack of power, but a lack of effect. The assessment of PUFA exposure at only one time point cannot capture PUFA status changes throughout follow-up. Finally, the inability to rule out the possibility of residual or unmeasured confounding also precludes inferences about causality [29].

In conclusion, we found no meaningful relationship between RBC levels of n-6 PUFAs and risk for CVD or total mortality. Importantly, after adjustment for covariates, there was no increase in risk for adverse outcomes with higher n-6 PUFA levels, with the possible exception of EDA. These findings, in the context of the totality of available evidence on this subject, provide no support for a harmful role of n-6 PUFAs in these important clinical outcomes.

## 5. Conclusions

Higher circulating levels of marine n-3 FA levels are associated with reduced risk for incident CVD and ischemic stroke and for death from CHD and all-causes; however, in the same sample little evidence exists for association with n-6 FAs. Further work is needed to identify a full profile of FAs associated with cardiovascular risk and mortality.

## Figures and Tables

**Table 1:** Demographic overview (*n* = 2500). METS: metabolic equivalent.

Variable	Died (*n* = 350)	Still Living (*n* = 2150)	Total	*p*-Value for Difference (Chi-Squared or *t*-Test)
	% (*n*) or Mean (SD)	% (*n*) or Mean (SD)	% (*n*) or Mean (SD)	
Sex				
Male	52.9% (185)	41.5% (893)	43.1% (1078)	<0.001
Female	47.1% (165)	58.5% (1257)	56.9% (1422)	
Age	72.9 (8.6)	64.4 (8.2)	65.56 (8.76)	<0.001
Body Mass Index (BMI)	27.9 (5.9)	28.3 (5.4)	28.2 (5.4)	<0.001
Marital Status				
Single/Never Married	4.3% (15)	6.7% (144)	6.4% (159)	<0.001
Married	63.7% (223)	70.7% (1519)	69.7% (1742)	
Separated/Divorced	12.0% (42)	12.7% (273)	12.6% (315)	
Widowed	18.3% (64)	9.4% (203)	10.7% (267)	
Education				
Some High School (HS) or less	6.0% (21)	2.4% (51)	2.9% (72)	<0.001
HS graduate	33.7% (118)	24.8% (533)	26.0% (651)	
Some college or vocational	22.9% (78)	21.9% (471)	22.0% (549)	
College graduate	36.6% (128)	50.5% (1086)	48.6% (1214)	
Employment				
Employed	31.1% (109)	56.3% (1210)	52.8% (1319)	<0.001
Disabled/unemployed	3.1% (11)	2.5% 53)	2.6% (64)	
Retired	64.3% (225)	40.8% (877)	44.1% (1102)	
Health insurance status				
No insurance	2.3% (8)	1.9% (40)	1.9% (48)	0.053
Insurance, but no prescription	11.4% (40)	8.4% (181)	8.8% (221)	
Full insurance	84.0% (294)	88.7% (1906)	88.0% (2200)	
Regular aspirin use	49.7% (174)	38.6% (830)	40.2% (1004)	<0.001
Prevalent hypertension	57.1% (200)	42.2% (908)	44.3% (1108)	<0.001
Cholesterol medication	41.4% (145)	36.9% (794)	37.6% (939)	0.06
Prevalent diabetes	22.3% (78)	11.6% (249)	13.1% (327)	<0.001
Alcohol consumption				
None	34.0% (119)	23.2% (499)	24.7% (618)	<0.001
<1 drink/day	36.0% (126)	50.7% (1089)	48.6% (1215)	
1–2 drinks/day	22.6% (79)	20.2% (434)	20.5% (513)	
>2 drinks/day	6.9% (24)	5.7% (123)	5.9% (147)	
Smoking				
Not current smoker	89.1% (312)	90.5% (1946)	90.3% (2258)	0.70
Current	10.6% (37)	9.3% (200)	9.5% (237)	
METS	4.7 (15.4)	3.3 (8.6)	3.5 (9.9)	<0.001
Total to High-Density Lipoprotein (HDL) cholesterol ratio	3.5 (1.1)	3.5 (1.0)	3.5 (1.1)	<0.001
Systolic Blood Pressure	133.6 (19.8)	128.2 (16.9)	129.0 (17.4)	<0.001
C-reactive protein	4.9 (12.7)	3.0 (6.1)	3.3 (7.4)	<0.001
Omega-3 index	0.054 (0.017)	0.056 (0.017)	0.0554 (0.0168)	<0.001

**Table:** 

(a)							
	**Total Events**			**Mortality**			
	**CVD**	**CHD**	**Stroke**	**CVD**	**Cancer**	**Other**	**Total**
	**HR (95% CI)**	**HR (95% CI)**	**HR (95% CI)**	**HR (95% CI)**	**HR (95% CI)**	**HR (95% CI)**	**HR (95% CI)**
A. Unadjusted							
<9.8% 18-carbon (*n* = 433)	1.0	1.0	1.0	1.0	1.0	1.0	1.0
9.8%–10.7% (*n* = 488)	1.07 (0.71, 1.61)	1.30 (0.72, 2.35)	1.28 (0.67, 2.43)	1.14 (0.45, 2.89)	0.96 (0.58, 1.58)	0.56 (0.31, 1.02)	0.92 (0.64, 1.31)
10.7%–11.5% (*n* = 522)	1.05 (0.71, 1.57)	1.08 (0.59, 1.98)	1.33 (0.72, 2.43)	0.86 (0.33, 2.19)	0.81 (0.48, 1.36)	0.51 (0.29, 0.92) *	0.77 (0.54, 1.11)
11.5%–12.5% (*n* = 522)	0.74 (0.48, 1.13)	0.80 (0.42, 1.52)	0.70 (0.35, 1.38)	1.74 (0.71, 4.25)	0.56 (0.32, 1.00)	0.86 (0.53, 1.39)	0.94 (0.67, 1.31)
>12.5% (*n* = 535)	0.73 (0.48, 1.13)	1.01 (0.56, 1.81)	0.80 (0.39, 1.63)	0.57 (0.22, 1.53)	0.74 (0.43, 1.25)	0.52 (0.29, 0.93) *	0.74 (0.52, 1.06)
*p*-value for linear trend	0.032 *	0.45	0.15	0.26	0.08	0.17	0.14
B. Adjusted for demos							
<9.8% 18-carbon (*n* = 433)	1.0	1.0	1.0	1.0	1.0	1.0	1.0
9.8%–10.7% (*n* = 488)	1.28 (0.83, 1.95)	1.42 (0.76, 2.67)	1.46 (0.71, 3.00)	1.19 (0.48, 2.96)	1.09 (0.63, 1.88)	0.87 (0.47, 1.60)	1.21 (0.84, 1.73)
10.7%–11.5% (*n* = 522)	1.37 (0.90, 2.08)	1.38 (0.74, 2.56)	1.51 (0.77, 2.96)	1.12 (0.42, 2.97)	0.89 (0.49, 1.61)	0.83 (0.45, 1.56)	1.02 (0.69, 1.50)
11.5%–12.5% (*n* = 522)	0.78 (0.48, 1.27)	0.96 (0.46, 2.02)	0.72 (0.33, 1.61)	1.59 (0.67, 3.75)	0.65 (0.35, 1.20)	1.02 (0.58, 1.80)	0.99 (0.68, 1.44)
>12.5% (*n* = 535)	1.16 (0.72, 1.89)	1.53 (0.75, 3.13)	1.12 (0.52, 2.40)	0.96 (0.33, 2.80)	0.88 (0.47, 1.65)	0.62 (0.29, 1.33)	1.02 (0.68, 1.53)
*p*-value for linear trend	0.68	0.59	0.49	0.68	0.30	0.42	0.69
C. Adjusted for demos and O3I							
<9.8% 18-carbon (*n* = 433)	1.0	1.0	1.0	1.0	1.0	1.0	1.0
9.8%–10.7% (*n* = 488)	1.19 (0.78, 1.82)	1.32 (0.70, 2.49)	1.29 (0.62, 2.66)	1.08 (0.43, 2.73)	1.07 (0.62, 1.87)	0.74 (0.39, 1.41)	1.14 (0.79, 1.64)
10.7%–11.5% (*n* = 522)	1.28 (0.84, 1.95)	1.28 (0.69, 2.38)	1.34 (0.67, 2.65)	1.04 (0.38, 2.78)	0.87 (0.47, 1.61)	0.71 (0.38, 1.34)	0.95 (0.64, 1.41)
11.5%–12.5% (*n* = 522)	0.71 (0.44, 1.16)	0.86 (0.41, 1.79)	0.62 (0.28, 1.39)	1.49 (0.65, 3.44)	0.64 (0.34, 1.19)	0.83 (0.46, 1.50)	0.92 (0.63, 1.34)
>12.5% (*n* = 535)	1.03 (0.63, 1.67)	1.32 (0.64, 2.73)	0.93 (0.43, 2.03)	0.78 (0.26, 2.34)	0.86 (0.45, 1.63)	0.52 (0.24, 1.11)	0.92 (0.61, 1.40)
*p*-value for linear trend	0.34	0.92	0.23	0.87	0.28	0.18	0.38
*p*-value for omega-3	0.005 **	0.034 *	0.004 **	0.10	0.70	0.004 **	0.011

CVD, cardiovascular disease; CHD, coronary heart disease; CI, confidence interval; HR, hazard ratio; O3I, Omega-3 Index. * *p* < 0.05; ** *p* < 0.01. All significant hazard ratios/*p*-values are shown in bold italics. A. Unadjusted model, B. Adjusted for all variables in Table 1 except history of CVD, C. Adjusted for omega-3 index and all variables in Table 1 except history of CVD. Quintiles for 18-carbon n-6 fatty acids are shown in the left-most column. For example, 9.8%–10.7% means that the second quintile for RBC 18-carbon n-6 fatty acids are individuals for whom between 9.8 and 10.7% of all RBC fatty acids are 18-carbon n-6 fatty acids.

**Table:** 

(b)							
	**Total Events**			**Mortality**			
	**CVD**	**CHD**	**Stroke**	**CVD**	**Cancer**	**Other**	**Total**
	**HR (95% CI)**	**HR (95% CI)**	**HR (95% CI)**	**HR (95% CI)**	**HR (95% CI)**	**HR (95% CI)**	**HR (95% CI)**
A. Unadjusted							
<17.5% 20-carbon (*n* = 508)	1.0	1.0	1.0	1.0	1.0	1.0	1.0
17.5%–18.4% (*n* = 509)	1.03 (0.71, 1.50)	0.95 (0.53, 1.70)	0.96 (0.54, 1.70)	1.19 (0.54, 2.62)	0.80 (0.48, 1.32)	0.84 (0.49, 1.46)	0.89 (0.65, 1.22)
18.4%–19.1% (*n* = 502)	0.92 (0.61, 1.39)	0.95 (0.52, 1.71)	0.78 (0.41, 1.49)	0.83 (0.31, 2.20)	0.90 (0.53, 1.53)	1.59 (0.94, 2.67)	1.16 (0.83, 1.63)
19.1%–19.9% (*n* = 502)	0.88 (0.58, 1.33)	1.22 (0.71, 2.09)	0.63 (0.32, 1.24)	1.24 (0.55, 2.80)	0.87 (0.52, 1.46)	1.19 (0.67, 2.09)	1.02 (0.73, 1.42)
>19.9% (*n* = 479)	1.03 (0.70, 1.52)	0.99)0.55, 1.77)	1.04 (0.59, 1.82)	0.77 (0.37, 1.60)	0.92 (0.55, 1.53)	1.14 (0.65, 2.01)	0.95 (0.69, 1.33)
*p*-value for linear trend	0.82	0.71	0.68	0.54	0.83	0.32	0.90
B. Adjusted for demos							
<17.5% 20-carbon (*n* = 508)	1.0	1.0	1.0	1.0	1.0	1.0	1.0
17.5%–18.4% (*n* = 509)	1.10 (0.74, 1.65)	0.96 (0.52, 1.75)	1.19 (0.62, 2.30)	1.58 (0.61, 4.11)	0.84 (0.49, 1.42)	0.86 (0.45, 1.67)	1.01 (0.71, 1.44)
18.4%–19.1% (*n* = 502)	0.96 (0.61, 1.51)	0.93 (0.49, 1.79)	1.09 (0.54, 2.21)	1.23 (0.38, 4.02)	0.98 (0.53, 1.81)	1.91 (1.08, 3.36) *	1.33 (0.92, 1.92)
19.1%–19.9% (*n* = 502)	1.04 (0.67, 1.61)	1.29 (0.71, 2.36)	0.88 (0.43, 1.80)	1.98 (0.75, 5.19)	1.02 (0.58 1.78)	1.45 (0.77, 2.70)	1.27 (0.88, 1.81)
>19.9% (*n* = 479)	1.21 (0.78, 1.88)	1.06 (0.56, 2.02)	1.48 (0.75, 2.92)	1.19 (0.44, 3.21)	1.21 (0.69, 2.13)	1.40 (0.75, 2.61)	1.19 (0.82, 1.73)
*p*-value for linear trend	0.52	0.52	0.53	0.55	0.42	0.11	0.16
C. Adjusted for demos and O3I							
<17.5% 20-carbon (*n* = 508)	1.0	1.0	1.0	1.0	1.0	1.0	1.0
17.5%–18.4% (*n* = 509)	0.97 (0.65, 1.46)	0.84 (0.46, 1.51)	0.96 (0.50, 1.86)	1.30 (0.47, 3.61)	0.84 (0.49, 1.44)	0.77 (0.40, 1.51)	0.94 (0.65, 1.36)
18.4%–19.1% (*n* = 502)	0.81 (0.51, 1.30)	0.77 (0.39, 1.51)	0.82 (0.39, 1.72)	0.99 (0.28, 3.55)	0.99 (0.52, 1.88)	1.62 (0.92, 2.86)	1.19 (0.81, 1.75)
19.1%–19.9% (*n* = 502)	0.85 (0.53, 1.34)	1.03 (0.55, 1.91)	0.61 (0.28, 1.33)	1.55 (0.56, 4.30)	1.03 (0.57, 1.86)	1.17 (0.60, 2.27)	1.12 (0.76, 1.65)
>19.9% (*n* = 479)	0.97 (0.60, 1.56)	0.82 (0.41, 1.65)	1.03 (0.49, 2.15)	0.85 (0.27, 2.72)	1.23 (0.67, 2.26)	1.12 (0.58, 2.16)	1.04 (0.70, 1.56)
*p*-value for linear trend	0.72	0.87	0.91	0.89	0.42	0.44	0.58
*p*-value for omega-3	0.011 *	0.039 *	0.009 **	0.13	0.87	0.03 *	0.04 *

CVD, cardiovascular disease; CHD, coronary heart disease; CI, confidence interval; HR, hazard ratio; O3I, Omega-3 Index. * *p* < 0.05; ** *p* < 0.01. All significant hazard ratios/*p*-values are shown in bold italics. A. Unadjusted model, B. Adjusted for all variables in Table 1 except history of CVD, C. Adjusted for omega-3 index and all variables in Table 1 except history of CVD. Quintiles for 18-carbon n-6 fatty acids are shown in the left-most column. For example, 17.5%–18.4% means that the second quintile for RBC 20-carbon n-6 fatty acids are individuals for whom between 17.5 and 18.4% of all RBC fatty acids are 20-carbon n-6 fatty acids.

**Table:** 

(c)							
	**Total Events**			**Mortality**			
	**CVD**	**CHD**	**Stroke**	**CVD**	**Cancer**	**Other**	**Total**
	**HR (95% CI)**	**HR (95% CI)**	**HR (95% CI)**	**HR (95% CI)**	**HR (95% CI)**	**HR (95% CI)**	**HR (95% CI)**
A. Unadjusted							
<3.6% 22-carbon (*n* = 508)	1.0	1.0	1.0	1.0	1.0	1.0	1.0
3.6%–4.3% (*n* = 508)	1.37 (0.88, 2.12)	1.24 (0.63, 2.43)	1.25 (0.62, 2.53)	0.93 (0.42, 2.03)	0.86 (0.52, 1.42)	1.11 (0.64, 1.93)	0.96 (0.69, 1.31)
4.3%–4.7% (*n* = 500)	1.73 (1.14, 2.62) **	2.43 (1.34, 4.42) **	1.36 (0.71, 2.61)	0.81 (0.35, 1.83)	0.65 (0.38, 1.13)	0.96 (0.53, 1.73)	0.80 (0.57, 1.13)
4.7%–5.3% (*n* = 509)	1.46 (0.96, 2.22)	1.61 (0.86, 3.03)	1.58 (0.83, 2.98)	1.16 (0.54, 2.49)	0.78 (0.46, 1.31)	1.27 (0.72, 2.26)	1.04 (0.75, 1.45)
>5.3% (*n* = 469)	1.73 (1.12, 2.68) *	2.25 (1.22, 4.16) **	1.48 (0.74, 2.98)	0.63 (0.24, 1.67)	1.18 (0.71, 1.95)	1.43 (0.82, 2.50)	1.10 (0.77, 1.56)
*p*-value for linear trend	0.02 *	0.006 **	0.18	0.57	0.74	0.17	0.48
B. Adjusted for demos							
<3.6% 22-carbon (*n* = 508)	1.0	1.0	1.0	1.0	1.0	1.0	1.0
3.6%–4.3% (*n* = 508)	1.20 (0.75, 1.93)	1.05 (0.50, 2.20)	1.39 (0.62, 3.13)	0.83 (0.28, 2.41)	0.94 (0.56, 1.60)	1.06 (0.60, 1.88)	1.01 (0.71, 1.44)
4.3%–4.7% (*n* = 500)	1.74 (1.13, 2.67) *	2.38 (1.26,4.48) **	1.73 (0.85, 3.55)	0.65 (0.26, 1.62)	0.74 (0.41, 1.32)	0.85 (0.41, 1.76)	0.85 (0.58, 1.25)
4.7%–5.3% (*n* = 509)	1.36 (0.87, 2.13)	1.51 (0.76, 2.99)	1.89 (0.92, 3.87)	1.28 (0.62, 2.62)	0.74 (0.41, 1.31)	1.31 (0.68, 2.51)	1.11 (0.78, 1.59)
>5.3% (*n* = 469)	1.66 (1.02, 2.69) *	1.91 (0.95, 3.83)	2.01 (0.92, 4.42)	0.60 (0.18, 2.01)	1.21 (0.71, 2.07)	1.53 (0.85, 2.74)	1.17 (0.81, 1.71)
*p*-value for linear trend	0.031 *	0.04 *	0.04 *	0.80	0.91	0.10	0.32
C. Adjusted for demos and O3I							
<3.6% 22-carbon (*n* = 508)	1.0	1.0	1.0	1.0	1.0	1.0	1.0
3.6%–4.3% (*n* = 508)	1.11 (0.69, 1.79)	0.99 (0.48, 2.05)	1.20 (0.53, 2.69)	0.62 (0.20, 1.95)	0.93 (0.54, 1.60)	0.89 (0.48, 1.63)	0.89 (0.62, 1.29)
4.3%–4.7% (*n* = 500)	1.52 (0.96, 2.39)	2.09 (1.07, 4.06) *	1.35 (0.65, 2.80)	0.42 (0.17, 1.06)	0.72 (0.38, 1.36)	0.67 (0.32, 1.42)	0.69 (0.45, 1.05)
4.7%–5.3% (*n* = 509)	1.14 (0.70, 1.86)	1.25 (0.60, 2.62)	1.37 (0.64, 2.97)	0.69 (0.27, 1.76)	0.71 (0.38, 1.34)	0.97 (0.48, 1.94)	0.87 (0.58, 1.30)
>5.3% (*n* = 469)	1.33 (0.77, 2.28)	1.51 (0.69, 3.30)	1.38 (0.60, 3.18)	0.30 (0.07, 1.26)	1.15 (0.57, 2.33)	0.98 (0.48, 1.98)	0.85 (0.54, 1.34)
*p*-value for linear trend	0.42	0.34	0.44	0.14	0.97	0.88	0.57
*p*-value for omega-3	0.09	0.22	0.049 *	0.034 *	0.92	0.03 *	0.021 *

CVD, cardiovascular disease; CHD, coronary heart disease; CI, confidence interval; HR, hazard ratio; O3I, Omega-3 Index. * *p* < 0.05; ** *p* < 0.01. All significant hazard ratios/*p*-values are shown in bold italics. A. Unadjusted model, B. Adjusted for all variables in Table 1 except history of CVD, C. Adjusted for omega-3 index and all variables in Table 1 except history of CVD. Quintiles for 18-carbon n-6 fatty acids are shown in the left-most column. For example, 3.6%–4.3% means that the second quintile for RBC 22-carbon n-6 fatty acids are individuals for whom between 3.6 and 4.3% of all RBC fatty acids are 22-carbon n-6 fatty acids.

## Supplementary Materials

The following are available online at http://www.mdpi.com/2072-6643/10/12/2012/s1, Table S1: Correlation between and among red blood cell n-3 and n-6 fatty acids, Table S2: Distribution of outcomes (*n* = 2500), Table S3: Risk of events and mortality by individual n-6 fatty acids (*n* = 2500), Table S3a: Linoleic Acid—Unadjusted, Adjusted for demographics (Table 1 variables), and further adjusted for the Omega-3 Index (5-groups), Table S3b: Gamma-linolenic Acid, Table S3c: Eicosadienoic Acid, Table S3d: Eicosatrienoic Acid, Table S3e: Arachidonic Acid, Table S3f: Docosatetraenoic Acid, Table S3g: Docosapentaenoic Acid, Table S3h: n6 index (sum of all 7 n6’s).

## Abbreviations

ADA	adrenic acid
AA	arachidonic acid
CVD	cardiovascular disease
DGLA	dihomo-gamma linolenic acid
DHA	docosahexaenoic acid
DPA n-6	docosapentaenoic acid
EDA	eicosadienoic acid
EPA	eicosapentaenoic acid
FA	fatty acid
GLA	gamma-linolenic acid
HR	hazard ratio
LA	linoleic acid
MI	myocardial infarction
Omega-3 Index	erythrocyte EPA + DHA
PUFA	polyunsaturated FA
RBC	red blood cell
SD	standard deviation
