# Supplementary material for: Erythrocyte n-6 Fatty Acids and Risk for Cardiovascular Outcomes and Total Mortality in the Framingham Heart Study
Source: Nutrients. 2018 Dec 19;10(12):2012. doi: 10.3390/nu10122012 (PMC6316092; doi:10.3390/nu10122012)
Supplement: Supplementary file 1 [file nutrients-10-02012-s001.pdf]

Supplemental Tables

Table S1. Correlation between and among red blood cell n-3 and n-6 fatty acids

	18:3n3	20:5n3	22:5n3	22:6n3	18:2n6	18:3n6	20:2n6	20:3n6	20:4n6	22:4n6	22:5n6
18:3n3	1.00	0.11	0.03	-0.01	0.24	0.01	0.13	0.07	-0.23	-0.22	-0.17
20:5n3		1.00	0.64	0.65	-0.13	-0.02	-0.20	-0.20	-0.46	-0.65	-0.63
22:5n3			1.00	0.42	-0.32	-0.10	-0.17	-0.16	-0.21	-0.25	-0.40
22:6n3				1.00	-0.26	-0.07	-0.09	-0.21	-0.33	-0.58	-0.48
18:2n6					1.00	0.05	0.43	0.26	-0.47	-0.23	-0.17
18:3n6						1.00	0.02	0.02	-0.02	-0.03	-0.04
20:2n6							1.00	0.26	-0.22	0.05	-0.01
20:3n6								1.00	-0.32	-0.04	0.15
20:4n6									1.00	0.57	0.49
22:4n6										1.00	0.66
22:5n6											1.00

Table S2. Distribution of outcomes (n=2500)

	Number of people with events (cases)	Number of people without events (controls)	Median follow-up days[1]	Maximum follow-up days[1]
Total CVD	245	2255	2351	3833
Total CHD	119	2381	2342	3815
Ischemic stroke	105	2395	2342	3833
CVD mortality	58	2442	2673	3815
Death from any cause[2]	350	2150	2686	3815

1. Across all 2500 participants and so computed as days to event or days to censoring
2. The causes of the 350 observed deaths, besides CVD, were 146 Cancer (42%), 128 Other (37%) and 18 Unknown (5%).

Table S3. Risk of events and mortality by individual n-6 fatty acids (n=2500)

Table S3a. Linoleic Acid – Unadjusted, Adjusted for demographics (Table 1 variables), and further adjusted for the Omega-3 Index (5-groups)

	Hazard ratios (95% CIs)						
	Total Events			Mortality			
**A. Linoleic (18:2n6)**	**CVD**	**CHD**	**Stroke**	**CVD**	**Cancer**	**Other**	**Total**
**<9.7% (n=436)**	1.0	1.0	1.0	1.0	1.0	1.0	1.0
**9.7-10.6% (n=486)**	1.12 (0.75, 1.70)	1.43 (0.79, 2.53)	1.30 (0.68, 2.46)	1.40 (0.55, 3.60)	0.92 (0.56, 1.52)	0.56 (0.31, 1.02)	0.92 (0.64, 1.31)
**10.6-11.4% (n=519)**	1.08 (0.72, 1.61)	1.19 (0.64, 2.20)	1.34 (0.73, 2.44)	0.86 (0.32, 2.28)	0.82 (0.49, 1.35)	***0.49 (0.27, 0.88)****	0.76 (0.53, 1.09)
**11.4-12.4% (n=522)**	0.79 (0.52, 1.21)	0.84 (0.44, 1.62)	0.75 (0.38, 1.46)	2.07 (0.83, 5.16)	***0.54 (0.30, 0.96)****	0.85 (0.53, 1.38)	0.94 (0.67, 1.31)
**>12.4% (n=537)**	0.72 (0.47, 1.11)	1.02 (0.55, 1.86)	0.76 (0.37, 1.56)	0.63 (0.23, 1.720	0.72(0.43, 1.22)	***0.51 (0.29, 0.91)****	0.74 (0.52, 1.05)
**p-value for linear trend**	***0.029****	0.41	0.13	0.77	0.073	0.14	0.14
	Hazard ratios (95% CIs)						
	Total Events			Mortality			
**B. Linoleic (18:2n6)**	**CVD**	**CHD**	**Stroke**	**CVD**	**Cancer**	**Other**	**Total**
**<9.7% (n=436)**	1.0	1.0	1.0	1.0	1.0	1.0	1.0
**9.7-10.6% (n=486)**	1.37 (0.89, 2.09)	1.57 (0.83, 2.96)	1.51 (0.73, 3.12)	1.57 (0.62, 3.97)	1.05 (0.61, 1.82)	0.86 (0.47, 1.56)	1.22 (0.85, 1.76)
**10.6-11.4% (n=519)**	1.42 (0.93, 2.16)	1.53 (0.81, 2.89)	1.51 (0.77, 2.97)	1.13 (0.40, 3.17)	0.91 (0.51, 1.63)	0.78 (0.42, 1.48)	1.01 (0.68, 1.49)
**11.4-12.4% (n=522)**	0.87 (0.54, 1.41)	1.03 (0.49, 2.18)	0.80 (0.37, 1.75)	2.12 (0.88, 5.10)	0.63 (0.34, 1.16)	1.00 (0.58, 1.76)	1.01 (0.69, 1.46)
**>12.4% (n=537)**	1.16 (0.71, 1.91)	1.56 (0.75, 3.25)	1.09 (0.49, 2.39)	1.11 (0.37, 3.32)	0.86 (0.46, 1.62)	0.61 (0.29, 1.28)	1.02 (0.67, 1.54)
**p-value for linear trend**	0.73	0.60	0.52	0.43	0.29	0.37	0.70
	Hazard ratios (95% CIs)						
	Total Events			Mortality			
**C. Linoleic (18:2n6)**	**CVD**	**CHD**	**Stroke**	**CVD**	**Cancer**	**Other**	**Total**
**<9.7% (n=436)**	1.0	1.0	1.0	1.0	1.0	1.0	1.0
**9.7-10.6% (n=486)**	1.27 (0.83, 1.95)	1.46 (0.77, 2.75)	1.32 (0.64, 2.74)	1.43 (0.56, 3.66)	1.04 (0.59, 1.81)	0.72 (0.38, 1.36)	1.15 (0.80, 1.66)
**10.6-11.4% (n=519)**	1.31 (0.86, 2.01)	1.41 (0.75, 2.67)	1.33 (0.67, 2.65)	1.04 (0.37, 2.95)	0.89 (0.49, 1.63)	0.67 (0.35, 1.26)	0.94 (0.63, 1.40)
**11.4-12.4% (n=522)**	0.79 (0.49, 1.28)	0.92 (0.44, 1.95)	0.68 (0.31, 1.51)	1.97 (0.84, 4.62)	0.61 (0.32, 1.16)	0.82 (0.46, 1.45)	0.93 (0.64, 1.35)
**>12.4% (n=537)**	1.03 (0.62, 1.70)	1.35 (0.65, 2.84)	0.90 (0.40, 2.02)	0.91 (0.30, 2.79)	0.84 (0.44, 1.60)	0.50 (0.25, 1.07)	0.92 (0.61, 1.40)
**p-value for linear trend**	0.38	0.93	0.25	0.59	0.26	0.16	0.39
**p-value for omega-3**	***0.005*****	***0.033****	***0.004*****	0.11	0.69	***0.004*****	***0.011****

CVD, cardiovascular disease; CHD, coronary heart disease; CI, confidence interval.

*P<0.05; **P<0.01

All significant hazard ratios/P-values are shown in bold italics.

A. Unadjusted model, B. Adjusted for all variables in Table 1 except history of CVD, C. Adjusted for omega-3 index and all variables in Table 1 except history of CVD

Table S3b. Gamma-linolenic Acid

	Hazard ratios (95% CIs)						
	**Total Events**			**Mortality**			
**A. Gamma-linolenic (18:3n6)**	**CVD**	**CHD**	**Stroke**	**CVD**	**Cancer**	**Other**	**Total**
**<0.04% (n=500)**	1.0	1.0	1.0	1.0	1.0	1.0	1.0
**0.04-0.06% (n=497)**	0.79 (0.53, 1.18)	0.76 (0.41, 1.40)	0.72 (0.40, 1.32)	0.60 (0.26, 1.35)	0.87 (0.52, 1.46)	0.63 (0.35, 1.12)	***0.68 (0.49, 0.96)****
**0.06-0.07% (n=495)**	0.96 (0.64, 1.46)	1.50 (0.87, 2.58)	0.49 (0.24, 1.00)	0.95 (0.42, 2.17)	0.79 (0.47, 1.34)	0.82 (0.48, 1.40)	0.79 (0.56, 1.11)
**0.07-0.10% (n=504)**	0.93 (0.63, 1.36)	0.91 (0.49, 1.67)	0.96 (0.57, 1.64)	0.66 (0.27, 1.59)	0.95 (0.56, 1.60)	0.59 (0.32, 1.10)	0.74 (0.52, 1.05)
**>0.10% (n=504)**	0.92 (0.62, 1.37)	1.04 (0.58, 1.86)	0.82 (0.44, 1.52)	1.22 (0.58, 2.57)	0.86 (0.50, 1.47)	0.97 (0.56, 1.10)	0.95 (0.69, 1.32)
**p-value for linear trend**	0.98	0.70	0.83	0.46	0.72	0.83	0.95
	**Hazard ratios (95% CIs)**						
	**Total Events**			**Mortality**			
**B. Gamma-linolenic (18:3n6)**	**CVD**	**CHD**	**Stroke**	**CVD**	**Cancer**	**Other**	**Total**
**<0.04% (n=500)**	1.0	1.0	1.0	1.0	1.0	1.0	1.0
**0.04-0.06% (n=497)**	0.79 (0.51, 1.23)	0.66 (0.34, 1.32)	0.75 (0.38, 1.48)	1.15 (0.40, 3.24)	1.01 (0.57, 1.79)	0.67 (0.33, 1.34)	0.81 (0.55, 1.18)
**0.06-0.07% (n=495)**	1.04 (0.67, 1.60)	1.48 (0.82, 2.65)	0.60 (0.29, 1.28)	1.60 (0.59, 4.35)	0.96 (0.54, 1.70)	0.86 (0.47, 1.55)	0.94 (0.65, 1.35)
**0.07-0.10% (n=504)**	1.01 (0.66, 1.54)	0.88 (0.43, 1.80)	1.07 (0.57, 2.01)	0.93 (0.30, 2.93)	1.07 (0.58, 2.00)	***0.42 (0.21, 0.81)*****	0.79 (0.53, 1.16)
**>0.10% (n=504)**	0.89 (0.58, 1.35)	0.92 (0.49, 1.71)	0.81 (0.41, 1.61)	1.78 (0.73, 4.35)	1.02 (0.57, 1.83)	0.84 (0.45, 1.55)	1.07 (0.75, 1.53)
**p-value for linear trend**	0.97	0.85	0.91	0.27	0.87	0.32	0.74
	**Hazard ratios (95% CIs)**						
	**Total Events**			**Mortality**			
**C. Gamma-linolenic (18:3n6)**	**CVD**	**CHD**	**Stroke**	**CVD**	**Cancer**	**Other**	**Total**
**<0.04% (n=500)**	1.0	1.0	1.0	1.0	1.0	1.0	1.0
**0.04-0.06% (n=497)**	0.74 (0.48, 1.14)	0.62 (0.31, 1.22)	0.68 (0.35, 1.35)	1.02 (0.36, 2.89)	1.01 (0.56, 1.79)	0.58 (0.29, 1.16)	0.75 (0.51, 1.10)
**0.06-0.07% (n=495)**	0.99 (0.64, 1.52)	1.39 (0.78, 2.49)	0.58 (0.27, 1.21)	1.55 (0.57, 4.19)	0.96 (0.54, 1.70)	0.75 (0.41, 1.37)	0.90 (0.62, 1.29)
**0.07-0.10% (n=504)**	0.94 (0.62, 1.43)	0.83 (0.41, 1.70)	0.96 (0.51, 1.80)	0.82 (0.26, 2.54)	1.07 (0.57, 2.00)	***0.37 (0.19, 0.73)*****	0.73 (0.50, 1.08)
**>0.10% (n=504)**	0.80 (0.53, 1.22)	0.83 (0.45, 1.52)	0.71 (0.35, 1.42)	1.52 (0.63, 3.66)	1.02 (0.57, 1.82)	0.69 (0.37, 1.30)	0.97 (0.68, 1.38)
**p-value for linear trend**	0.73	0.93	0.66	0.39	0.89	0.16	0.93
**p-value for omega-3**	***0.008*****	***0.03****	***0.005*****	0.12	0.89	***0.004*****	***0.014****

CVD, cardiovascular disease; CHD, coronary heart disease; CI, confidence interval.

*P<0.05; **P<0.01

All significant hazard ratios/P-values are shown in bold italics.

A. Unadjusted model, B. Adjusted for all variables in Table 1 except history of CVD, C. Adjusted for omega-3 index and all variables in Table 1 except history of CVD

Table S3c. Eicosadienoic Acid

	Hazard ratios (95% CIs)						
	Total Events			Mortality			
**A. Eicosadienoic (20:2n6)**	**CVD**	**CHD**	**Stroke**	**CVD**	**Cancer**	**Other**	**Total**
**<0.24% (n=482)**	1.0	1.0	1.0	1.0	1.0	1.0	1.0
**0.24-0.26% (n=499)**	0.91 (0.61, 1.36)	0.86 (0.48, 1.53)	1.03 (0.54, 1.97)	0.61 (0.24, 1.53)	1.31 (0.77, 2.23)	1.11 (0.65, 1.88)	1.14 (0.81, 1.62)
**0.26-0.28% (n=502)**	0.76 (0.49, 1.18)	0.56 (0.29, 1.08)	1.12 (0.57, 2.18)	0.50 (0.17, 1.44)	0.93 (0.53, 1.66)	0.95 (0.52, 1.70)	0.97 (0.67, 1.41)
**0.28-0.31% (n=506)**	1.07 (0.73, 1.56)	0.79 (0.44, 1.43)	1.32 (0.72, 2.43)	1.20 (0.52, 2.79)	1.23 (0.72, 2.11)	1.13 (0.67, 1.93)	1.32 (0.95, 1.85)
**>0.31% (n=511)**	1.07 (0.72, 1.60)	1.32 (0.80, 2.17)	1.02 (0.53, 1.97)	1.35 (0.52, 2.79)	1.32 (0.78, 2.23)	1.25 (0.73, 2.14)	1.37 (0.98, 1.92)
**p-value for linear trend**	0.72	0.34	0.67	0.19	0.37	0.44	***0.035****
	Hazard ratios (95% CIs)						
	Total Events			Mortality			
**B. Eicosadienoic (20:2n6)**	**CVD**	**CHD**	**Stroke**	**CVD**	**Cancer**	**Other**	**Total**
**<0.24% (n=482)**	1.0	1.0	1.0	1.0	1.0	1.0	1.0
**0.24-0.26% (n=499)**	0.91 (0.59, 1.40)	0.75 (0.40, 1.40)	1.12 (0.53, 2.35)	0.47 (0.17, 1.27)	1.57 (0.91, 2.72)	1.06 (0.55, 2.04)	1.24 (0.84, 1.83)
**0.26-0.28% (n=502)**	0.89 (0.56, 1.41)	0.67 (0.34, 1.34)	1.20 (0.58, 2.48)	0.43 (0.14, 1.31)	0.98 (0.50, 1.92)	1.12 (0.61, 2.02)	1.12 (0.76, 1.66)
**0.28-0.31% (n=506)**	1.10 (0.71, 1.71)	0.99 (0.51, 1.92)	1.25 (0.62, 2.50)	0.80 (0.28, 2.24)	1.28 (0.71, 2.31)	1.20 (0.68, 2.14)	1.28 (0.88, 1.85)
**>0.31% (n=511)**	1.26 (0.82, 1.92)	1.64 (0.92, 2.93)	1.16 (0.59, 2.28)	1.34 (0.56, 3.21)	1.46 (0.83, 2.56)	1.46 (0.82, 2.60)	***1.52 (1.06, 2.17)****
**p-value for linear trend**	0.18	0.07	0.60	0.33	0.35	0.18	***0.028****
	Hazard ratios (95% CIs)						
	Total Events			Mortality			
**C. Eicosadienoic (20:2n6)**	**CVD**	**CHD**	**Stroke**	**CVD**	**Cancer**	**Other**	**Total**
**<0.24% (n=482)**	1.0	1.0	1.0	1.0	1.0	1.0	1.0
**0.24-0.26% (n=499)**	0.87 (0.56, 1.34)	0.72 (0.38, 1.34)	1.05 (0.49, 2.24)	0.44 (0.16, 1.23)	1.57 (0.90, 2.72)	0.98 (0.50, 1.92)	1.21 (0.82, 1.80)
**0.26-0.28% (n=502)**	0.85 (0.53, 1.35)	0.64 (0.32, 1.28)	1.12 (0.54, 2.33)	0.42 (0.13, 1.32)	0.98 (0.49, 1.94)	1.05 (0.57, 1.92)	1.09 (0.73, 1.61)
**0.28-0.31% (n=506)**	1.07 (0.69, 1.66)	0.97 (0.51, 1.87)	1.20 (0.61, 2.36)	0.85 (0.31, 2.36)	1.28 (0.70, 2.32)	1.09 (0.60, 1.99)	1.25 (0.85, 1.82)
**>0.31% (n=511)**	1.17 (0.76, 1.79)	1.52 (0.85, 2.70)	1.07 (0.54, 2.10)	1.25 (0.52, 3.00)	1.45 (0.82, 2.57)	1.34 (0.74, 2.41)	***1.46 (1.02, 2.10)****
**p-value for linear trend**	0.27	0.10	0.74	0.34	0.36	0.31	***0.045****
**p-value for omega-3**	***0.013****	0.051	***0.006*****	0.10	0.93	***0.013****	***0.023****

CVD, cardiovascular disease; CHD, coronary heart disease; CI, confidence interval.

*P<0.05; **P<0.01

All significant hazard ratios/P-values are shown in bold italics.

A. Unadjusted model, B. Adjusted for all variables in Table 1 except history of CVD, C. Adjusted for omega-3 index and all variables in Table 1 except history of CVD

Table S3d. Eicosatrienoic Acid

	Hazard ratios (95% CIs)						
	**Total Events**			**Mortality**			
**A. Eicosatrienoic (20:3n6)**	**CVD**	**CHD**	**Stroke**	**CVD**	**Cancer**	**Other**	**Total**
**<1.30% (n=489)**	1.0	1.0	1.0	1.0	1.0	1.0	1.0
**1.30-1.46% (n=477)**	1.10 (0.73, 1.65)	1.08 (0.58, 1.99)	1.26 (0.68, 2.32)	0.78 (0.32, 1.92)	1.10 (0.63, 1.93)	1.05 (0.62, 1.79)	1.01 (0.71, 1.43)
**1.46-1.63% (n=500)**	1.23 (0.82, 1.83)	1.24 (0.68, 2.26)	1.24 (0.68, 2.29)	1.00 (0.44, 2.27)	1.36 (0.80, 2.31)	0.88 (0.50, 1.56)	1.14 (0.81, 1.60)
**1.63-1.85% (n=504)**	1.12 (0.74, 1.69)	0.94 (0.50, 1.77)	1.06 (0.55, 2.04)	1.18 (0.53, 2.61)	1.03 (0.61, 1.77)	1.00 (0.59, 1.72)	1.02 (0.73, 1.44)
**>1.85% (n=530)**	0.94 (0.62, 1.41)	1.39 (0.79, 2.45)	0.71 (0.37, 1.35)	0.89 (0.42, 1.88)	1.16 (0.67, 2.02)	0.94 (0.52, 1.69)	1.03 (0.73, 1.44)
**p-value for linear trend**	0.77	0.36	0.22	0.92	0.71	0.78	0.88
	**Hazard ratios (95% CIs)**						
	**Total Events**			**Mortality**			
**B. Eicosatrienoic (20:3n6)**	**CVD**	**CHD**	**Stroke**	**CVD**	**Cancer**	**Other**	**Total**
**<1.30% (n=489)**	1.0	1.0	1.0	1.0	1.0	1.0	1.0
**1.30-1.46% (n=477)**	1.22 (0.77, 1.91)	1.05 (0.54, 2.03)	1.52 (0.75, 3.08)	1.02 (0.32, 3.24)	1.12 (0.61, 2.06)	0.86 (0.47, 1.56)	1.05 (0.71, 1.56)
**1.46-1.63% (n=500)**	1.44 (0.93, 2.22)	1.19 (0.63, 2.25)	1.76 (0.90, 3.46)	1.40 (0.47, 4.20)	1.52 (0.84, 2.75)	1.00 (0.54, 1.82)	1.39 (0.96, 2.02)
**1.63-1.85% (n=504)**	1.31 (0.85, 2.04)	0.91 (0.46, 1.81)	1.65 (0.81, 3.33)	1.59 (0.57, 4.41)	1.07 (0.58, 1.98)	1.06 (0.60, 1.86)	1.21 (0.84, 1.76)
**>1.85% (n=530)**	1.05 (0.66, 1.67)	1.29 (0.69, 2.42)	0.90 (0.42, 1.94)	1.45 (0.49, 4.35)	1.37 (0.71, 2.66)	1.02 (0.56, 1.87)	1.37 (0.93, 2.01)
**p-value for linear trend**	0.72	0.58	0.78	0.32	0.43	0.69	0.083
	**Hazard ratios (95% CIs)**						
	**Total Events**			**Mortality**			
**C. Eicosatrienoic (20:3n6)**	**CVD**	**CHD**	**Stroke**	**CVD**	**Cancer**	**Other**	**Total**
**<1.30% (n=489)**	1.0	1.0	1.0	1.0	1.0	1.0	1.0
**1.30-1.46% (n=477)**	1.18 (0.75, 1.85)	1.00 (0.52, 1.94)	1.47 (0.73, 2.98)	1.04 (0.34, 3.19)	1.12 (0.61, 2.07)	0.82 (0.45, 1.50)	1.04 (0.71, 1.54)
**1.46-1.63% (n=500)**	1.37 (0.89, 2.11)	1.12 (0.59, 2.13)	1.64 (0.84, 3.20)	1.32 (0.43, 4.00)	1.52 (0.83, 2.79)	0.92 (0.49, 1.71)	1.32 (0.90, 1.94)
**1.63-1.85% (n=504)**	1.25 (0.80, 1.93)	0.85 (0.43, 1.67)	1.53 (0.76, 3.09)	1.51 (0.55, 4.15)	1.07 (0.58, 1.99)	1.00 (0.57, 1.76)	1.18 (0.82, 1.71)
**>1.85% (n=530)**	0.95 (0.59, 1.51)	1.15 (0.62, 2.15)	0.75 (0.34, 1.65)	1.27 (0.44, 3.69)	1.38 (0.71, 2.68)	0.87 (0.46, 1.63)	1.27 (0.86, 1.88)
**p-value for linear trend**	0.89	0.85	0.57	0.46	0.43	0.94	0.18
**p-value for omega-3**	***0.009*****	***0.032****	***0.005*****	0.12	0.96	***0.009*****	***0.028****

CVD, cardiovascular disease; CHD, coronary heart disease; CI, confidence interval.

*P<0.05; **P<0.01

All significant hazard ratios/P-values are shown in bold italics.

A. Unadjusted model, B. Adjusted for all variables in Table 1 except history of CVD, C. Adjusted for omega-3 index and all variables in Table 1 except history of CVD

Table S3e. Arachidonic Acid

	Hazard ratios (95% CIs)						
	Total Events			Mortality			
**A. Arachidonic (20:4n6)**	**CVD**	**CHD**	**Stroke**	**CVD**	**Cancer**	**Other**	**Total**
**<15.6% (n=507)**	1.0	1.0	1.0	1.0	1.0	1.0	1.0
**15.6-16.5% (n=519)**	1.17 (0.80, 1.71)	1.01 (0.57, 1.77)	1.13 (0.63, 2.03)	***2.34 (1.10, 5.35)****	0.74 (0.45, 1.25)	1.13 (0.67, 1.92)	1.10 (0.80, 1.51)
**16.5-17.3% (n=502)**	0.93 (0.62, 1.39)	0.92 (0.52, 1.64)	0.77 (0.41, 1.47)	0.99 (0.39, 2.53)	0.82 (0.49, 1.36)	1.02 (0.57, 1.83)	0.92 (0.65, 1.30)
**17.3-18.1% (n=497)**	1.13 (0.75, 1.71)	1.23 (0.71, 2.13)	0.98 (0.52, 1.87)	1.68 (0.72, 3.93)	1.01 (0.61, 1.65)	1.28 (0.75, 2.20)	1.18 (0.86, 1.63)
**>18.1% (n=475)**	0.98 (0.66, 1.45)	0.87 (0.48, 1.57)	1.04 (0.58, 1.87)	0.84 (0.37, 1.88)	0.7 2(0.43, 1.21)	1.25 (0.72, 2.17)	0.92 (0.66, 1.28)
**p-value for linear trend**	0.86	0.96	0.95	0.42	0.53	0.35	0.83
	**Hazard ratios (95% CIs)**						
	**Total Events**			**Mortality**			
**B. Arachidonic (20:4n6)**	**CVD**	**CHD**	**Stroke**	**CVD**	**Cancer**	**Other**	**Total**
**<15.6% (n=507)**	1.0	1.0	1.0	1.0	1.0	1.0	1.0
**15.6-16.5% (n=519)**	1.29 (0.86, 1.94)	1.06 (0.58, 1.93)	1.44 (0.75, 2.79)	***3.11 (1.31, 7.31)*****	0.87 (0.51, 1.49)	1.34 (0.74, 2.38)	1.25 (0.89, 1.76)
**16.5-17.3% (n=502)**	0.94 (0.61, 1.42)	0.89 (0.48, 1.65)	0.97 (0.50, 1.87)	1.22 (0.30, 3.71)	0.80 (0.44, 1.47)	1.15 (0.60, 2.23)	1.04 (0.71, 1.52)
**17.3-18.1% (n=497)**	1.32 (0.86, 2.03)	1.37 (0.75, 2.51)	1.34 (0.67, 2.66)	2.45 (0.93, 6.46)	1.16 (0.68, 1.99)	1.50 (0.82, 2.74)	1.36 (0.95. 1.95)
**>18.1% (n=475)**	1.11 (0.71, 1.72)	0.93 (0.48, 1.77)	1.42 (0.73, 2.77)	1.08 (0.37, 3.19)	0.93 (0.53, 1.63)	1.51 (0.81, 2.80)	1.11 (0.76, 1.62)
**p-value for linear trend**	0.64	0.84	0.40	0.96	0.82	0.18	0.43
	**Hazard ratios (95% CIs)**						
	**Total Events**			**Mortality**			
**C. Arachidonic (20:4n6)**	**CVD**	**CHD**	**Stroke**	**CVD**	**Cancer**	**Other**	**Total**
**<15.6% (n=507)**	1.0	1.0	1.0	1.0	1.0	1.0	1.0
**15.6-16.5% (n=519)**	1.17 (0.77, 1.75)	0.96 (0.53, 1.72)	1.24 (0.64, 2.39)	***2.69 (1.04, 6.99)****	0.86 (0.50, 1.50)	1.23 (0.68, 2.22)	1.18 (0.83, 1.67)
**16.5-17.3% (n=502)**	0.83 (0.54, 1.26)	0.76 (0.41, 1.40)	0.80 (0.41, 1.58)	1.05 (0.32, 2.48)	0.79 (0.42, 1.50)	0.99 (0.50, 1.95)	0.94 (0.63, 1.40)
**17.3-18.1% (n=497)**	1.12 (0.72, 1.75)	1.14 (0.62, 2.11)	1.03 (0.51, 2.09)	1.98 (0.66, 5.93)	1.15 (0.65, 2.02)	1.29 (0.69, 2.38)	1.21 (0.83, 1.78)
**>18.1% (n=475)**	0.93 (0.59, 1.47)	0.74 (0.38, 1.46)	1.09 (0.55, 2.19)	0.89 (0.28, 2.81)	0.92 (0.50, 1.68)	1.28 (0.68, 2.41)	0.99 (0.66, 1.47)
**p-value for linear trend**	0.69	0.62	0.97	0.54	0.85	0.46	0.95
**p-value for omega-3**	***0.009*****	***0.03****	***0.01****	0.09	0.94	***0.02****	***0.023****

CVD, cardiovascular disease; CHD, coronary heart disease; CI, confidence interval.

*P<0.05; **P<0.01

All significant hazard ratios/P-values are shown in bold italics.

A. Unadjusted model, B. Adjusted for all variables in Table 1 except history of CVD, C. Adjusted for omega-3 index and all variables in Table 1 except history of CVD

Table S3f. Docosatetraenoic Acid

	Hazard ratios (95% CIs)						
	**Total Events**			**Mortality**			
**A. Docosatetraenoic (22:4n6)**	**CVD**	**CHD**	**Stroke**	**CVD**	**Cancer**	**Other**	**Total**
**<3.08% (n=515)**	1.0	1.0	1.0	1.0	1.0	1.0	1.0
**3.08-3.62% (n=510)**	***1.60 (1.04, 2.46)****	1.62 (0.85, 3.08)	1.47 (0.71, 3.03)	1.25 (0.56, 2.78)	0.80 (0.49, 1.31)	1.29 (0.75, 2.23)	1.02 (0.75, 1.40)
**3.62-4.03% (n=502)**	***1.56 (1.02, 2.39)****	***2.19 (1.19, 4.03)****	1.38 (0.70, 2.74)	0.93 (0.40, 2.13)	0.61 (0.36, 1.05)	0.97 (0.53, 1.78)	0.78 (0.55, 1.09)
**4.03-4.47% (n=499)**	***1.57 (1.04, 2.37)****	1.58 (0.83, 3.00)	***1.98 (1.04, 3.77)****	1.23 (0.57, 2.4)	0.63 (0.37, 1.07)	1.14 (0.61, 2.12)	0.95 (0.68, 1.32)
**>4.47% (n=474)**	***1.68 (1.10, 2.58)****	***2.19 (1.18, 4.07)****	1.62 (0.80, 3.26)	0.68 (0.26, 1.78)	1.15 (0.71, 1.86)	1.43 (0.82, 2.49)	1.11 (0.78, 1.56)
**p-value for linear trend**	***0.036****	***0.025****	0.10	0.54	0.98	0.37	0.78
	**Hazard ratios (95% CIs)**						
	**Total Events**			**Mortality**			
**B. Docosatetraenoic (22:4n6)**	**CVD**	**CHD**	**Stroke**	**CVD**	**Cancer**	**Other**	**Total**
**<3.08% (n=515)**	1.0	1.0	1.0	1.0	1.0	1.0	1.0
**3.08-3.62% (n=510)**	1.42 (0.89, 2.26)	1.39 (0.70, 2.78)	1.75 (0.76, 4.00)	1.04 (0.39, 2.79)	0.78 (0.37, 1.31)	1.15 (0.63, 2.09)	0.97 (0.68, 1.37)
**3.62-4.03% (n=502)**	1.52 (0.98, 2.35)	2.01 (1.05, 3.84)*	1.80 (0.85, 3.82)	0.89 (0.36, 2.21)	0.70 (0.40, 1.21)	0.88 (0.42, 1.84)	0.85 (0.58, 1.24)
**4.03-4.47% (n=499)**	1.54 (1.00, 2.37)	1.45 (0.72, 2.92)	***2.51 (1.25, 5.04)*****	1.51 (0.71, 3.23)	0.56 (0.30, 1.04)	1.27 (0.66, 2.44)	1.00 (0.70, 1.42)
**>4.47% (n=474)**	1.56 (0.97, 2.50)	1.81 (0.89, 3.69)	2.11 (0.97, 4.59)	0.76 (0.25, 2.32)	1.15 (0.6, 1.93)	1.43 (0.78, 2.61)	1.14 (0.79, 1.66)
**p-value for linear trend**	0.068	0.13	***0.022****	0.92	0.91	0.22	0.49
	**Hazard ratios (95% CIs)**						
	**Total Events**			**Mortality**			
**C. Docosatetraenoic (22:4n6)**	**CVD**	**CHD**	**Stroke**	**CVD**	**Cancer**	**Other**	**Total**
**<3.08% (n=515)**	1.0	1.0	1.0	1.0	1.0	1.0	1.0
**3.08-3.62% (n=510)**	1.33 (0.84, 2.11)	1.30 (0.65, 2.59)	1.59 (0.70, 3.59)	0.94 (0.35, 2.55)	0.76 (0.45, 1.29)	0.99 (0.54, 1.82)	0.88 (0.62, 1.2)
**3.62-4.03% (n=502)**	1.30 (0.81, 2.07)	1.69 (0.84, 3.42)	1.44 (0.68, 3.07)	0.63 (0.24, 1.65)	0.66 (0.36, 1.20)	0.67 ((0.31, 1.45)	0.69 (0.46, 1.04)
**4.03-4.47% (n=499)**	1.26 (0.78, 2.03)	1.16 (0.55, 2.45)	1.92 (0.91, 4.06)	0.93 (0.36, 2.42)	0.52 (0.26, 1.02)	0.91 (0.46, 1.81)	0.77 (0.52, 1.15)
**>4.47% (n=474)**	1.24 (0.74, 2.08)	1.37 (0.61, 3.05)	1.58 (0.72, 3.48)	0.47 (0.13, 1.66)	1.05 (0.54, 2.06)	0.92 (0.47, 1.80)	0.84 (0.54, 1.29)
**p-value for linear trend**	0.64	0.71	0.27	0.29	0.81	0.77	0.39
**p-value for omega-3**	0.051	0.11	0.07	0.07	0.79	***0.02****	***0.012****

CVD, cardiovascular disease; CHD, coronary heart disease; CI, confidence interval.

*P<0.05; **P<0.01

All significant hazard ratios/P-values are shown in bold italics.

A. Unadjusted model, B. Adjusted for all variables in Table 1 except history of CVD, C. Adjusted for omega-3 index and all variables in Table 1 except history of CVD

Table S3g. Docosapentaenoic Acid

	Hazard ratios (95% CIs)						
	Total Events			Mortality			
**A. Docosapentaenoic (22:5n6)**	**CVD**	**CHD**	**Stroke**	**CVD**	**Cancer**	**Other**	**Total**
**<0.51% (n=493)**	1.0	1.0	1.0	1.0	1.0	1.0	1.0
**0.51-0.61% (n=504)**	1.41 (0.93, 2.12)	1.21 (0.65, 2.27)	***1.96 (1.01, 3.84)****	0.76 (0.35, 1.63)	0.69 (0.41, 1.16)	0.93 (0.49, 1.76)	0.79 (0.57, 1.11)
**0.61-0.70% (n=520)**	1.24 (0.81, 1.89)	1.28 (0.68, 2.41)	1.53 (0.78, 3.02)	0.59 (0.23, 1.53)	0.63 (0.37, 1.08)	1.57 (0.89, 2.77)	0.91 (0.65, 1.27)
**0.70-0.80% (n=497)**	1.41 (0.92, 2.16)	1.70 (0.95, 3.05)	1.26 (0.60, 3.02)	1.07 (0.47, 2.44)	0.72 (0.41, 1.22)	1.72 (0.97, 3.06)	1.08 (0.77, 1.51)
**>0.80% (n=486)**	1.44 (0.93, 2.22)	1.67 (0.92, 3.04)	1.64 (0.79, 3.40)	1.11 (0.50, 2.46)	1.01 (0.62, 1.65)	1.84 (1.03, 3.29)*	1.25 (0.89, 1.73)
**p-value for linear trend**	0.13	***0.04****	0.61	0.55	0.93	***0.006****	***0.048****
	Hazard ratios (95% CIs)						
	Total Events			Mortality			
**B. Docosapentaenoic (22:5n6)**	**CVD**	**CHD**	**Stroke**	**CVD**	**Cancer**	**Other**	**Total**
**<0.51% (n=493)**	1.0	1.0	1.0	1.0	1.0	1.0	1.0
**0.51-0.61% (n=504)**	1.31 (0.84, 2.03)	0.95 (0.47, 1.90)	***2.35 (1.10, 4.99)****	0.86 (0.35, 2.13)	0.77 (0.45, 1.33)	0.68 (0.35, 1.33)	0.81 (0.56, 1.17)
**0.61-0.70% (n=520)**	1.11 (0.70, 1.78)	1.15 (0.57, 2.34)	1.69 (0.79, 3.61)	0.45 (0.15, 1.32)	0.61 (0.34, 1.09)	1.26 (0.65, 2.46)	0.80 (0.55, 1.17)
**0.70-0.80% (n=497)**	1.46 (0.94, 2.27)	1.50 (0.79, 2.84)	1.85 (0.85, 4.01)	1.29 (0.54, 3.13)	0.78 (0.44, 1.39)	1.58 (0.87, 2.85)	1.12 (0.78, 1.62)
**>0.80% (n=486)**	1.39 (0.88, 2.20)	1.41 (0.73, 2.70)	2.01 (0.93, 4.35)	1.48 (0.61, 3.62)	1.04 (0.62, 1.76)	1.51 (0.83, 2.74)	1.28 (0.90, 1.80)
**p-value for linear trend**	0.13	0.30	0.23	0.25	0.89	***0.02****	***0.04****
	Hazard ratios (95% CIs)						
	Total Events			Mortality			
**C. Docosapentaenoic (22:5n6)**	**CVD**	**CHD**	**Stroke**	**CVD**	**Cancer**	**Other**	**Total**
**<0.51% (n=493)**	1.0	1.0	1.0	1.0	1.0	1.0	1.0
**0.51-0.61% (n=504)**	1.16 (0.73, 1.83)	0.85 (0.42, 1.72)	1.93 (0.91, 4.11)	0.63 (0.23, 1.76)	0.75 (0.43, 1.31)	0.57 (0.29, 1.14)	0.73 (0.50, 1.06)
**0.61-0.70% (n=520)**	0.92 (0.56, 1.50)	0.93 (0.45, 1.93)	1.23 (0.55, 2.73)	***0.27 (0.08, 0.88)****	0.58 (0.31, 1.08)	0.96 (0.47, 1.94)	0.67 (0.44, 1.00)
**0.70-0.80% (n=497)**	1.17 (0.72, 1.89)	1.17 (0.57, 2.40)	1.27 (0.56, 2.88)	0.83 (0.35, 2.05)	0.73 (0.38, 1.41)	1.20 (0.63, 2.32)	0.93 (0.63, 1.39)
**>0.80% (n=486)**	1.08 (0.64, 1.81)	1.06 (0.51, 2.20)	1.34 (0.57, 3.14)	0.89 (0.34, 2.33)	0.98 (0.54, 1.77)	1.09 (0.57, 2.11)	1.02 (0.69, 1.52)
**p-value for linear trend**	0.79	0.53	0.88	0.64	0.95	0.19	0.29
**p-value for omega-3**	***0.03****	0.11	***0.01****	0.15	0.92	0.07	0.09

CVD, cardiovascular disease; CHD, coronary heart disease; CI, confidence interval.

*P<0.05; **P<0.01

All significant hazard ratios/P-values are shown in bold italics.

A. Unadjusted model, B. Adjusted for all variables in Table 1 except history of CVD, C. Adjusted for omega-3 index and all variables in Table 1 except history of CVD

Table S3h. n6 index (sum of all 7 n6's)

	Hazard ratios (95% CIs)						
	**Total Events**			**Mortality**			
**A. Omega-6 index**	**CVD**	**CHD**	**Stroke**	**CVD**	**Cancer**	**Other**	**Total**
**<32.6% (n=473)**	1.0	1.0	1.0	1.0	1.0	1.0	1.0
**32.6-34.1% (n=502)**	1.01 (0.66, 1.54)	0.78 (0.39, 1.55)	1.03 (0.53, 2.00)	0.90 (0.38, 2.15)	0.79 (0.47, 1.33)	1.24 (0.74, 2.07)	1.08 (0.77, 1.51)
**34.1-35.1% (n=510)**	1.17 (0.79, 1.74)	1.52 (0.84, 2.75)	1.14 (0.62, 2.08)	1.25 (0.53, 2.94)	0.84 (0.52, 1.36)	1.06 (0.58, 1.94)	1.06 (0.75, 1.49)
**35.1-36.2% (n=491)**	***1.51 (1.01, 2.24)****	1.60 (0.90, 2.86)	1.61 (0.88, 2.94)	1.11 (0.52, 2.36)	0.79 (0.53, 1.38)	0.94 (0.54, 1.65)	1.05 (0.75, 1.45)
**>36.2% (n=524)**	0.77 (0.49, 1.20)	1.33 (0.74, 2.40)	0.47 (0.21, 1.07)	0.57 (0.22, 1.49)	0.64 (0.38, 1.10)	1.05 (0.61, 1.83)	0.86 (0.61, 1.21)
**p-value for linear trend**	0.93	0.06	0.42	0.52	0.19	0.76	0.41
	**Hazard ratios (95% CIs)**						
	**Total Events**			**Mortality**			
**B. Omega-6 index**	**CVD**	**CHD**	**Stroke**	**CVD**	**Cancer**	**Other**	**Total**
**<32.6% (n=473)**	1.0	1.0	1.0	1.0	1.0	1.0	1.0
**32.6-34.1% (n=502)**	0.94 (0.60, 1.48)	0.66 (0.30, 1.43)	1.08 (0.51, 2.30)	1.19 (0.38, 2.94)	0.86 (0.50, 1.49)	1.23 (0.68, 2.21)	1.15 (0.79, 1.66)
**34.1-35.1% (n=510)**	1.28 (0.84, 1.95)	1.68 (0.88, 3.21)	1.38 (0.70, 2.73)	2.16 (0.92, 5.10)	1.02 (0.61, 1.71)	1.48 (0.79, 2.77)	1.44 (1.00, 2.06)
**35.1-36.2% (n=491)**	***1.76 (1.17, 2.65)*****	1.82 (0.99, 3.36)	***2.31 (1.20, 4.47)****	1.81 (0.82, 3.96)	1.12 (0.65, 1.93)	0.96 (0.52, 1.77)	1.27 (0.89, 1.82)
**>36.2% (n=524)**	1.06 (0.65, 1.74)	1.69 (0.87, 3.26)	0.77 (0.32, 1.86)	1.05 (0.37, 2.97)	0.83 (0.44, 1.56)	1.72 (0.97, 3.07)	1.28 (0.87, 1.88)
**p-value for linear trend**	0.08	***0.006****	0.31	0.28	0.88	0.24	0.14
	**Hazard ratios (95% CIs)**						
	**Total Events**			**Mortality**			
**C. Omega-6 index**	**CVD**	**CHD**	**Stroke**	**CVD**	**Cancer**	**Other**	**Total**
**<32.6% (n=473)**	1.0	1.0	1.0	1.0	1.0	1.0	1.0
**32.6-34.1% (n=502)**	0.84 (0.52, 1.34)	0.63 (0.28, 1.40)	0.82 (0.36, 1.83)	1.01 (0.36, 2.78)	0.84 (0.48, 1.46)	0.95 (0.51, 1.78)	1.02 (0.70, 1.49)
**34.1-35.1% (n=510)**	1.03 (0.64, 1.66)	1.55 (0.79, 3.06)	0.85 (0.38, 1.91)	1.60 (0.59, 4.30)	0.97 (0.56, 1.66)	0.93 (0.45, 1.93)	1.17 (0.78, 1.76)
**35.1-36.2% (n=491)**	1.35 (0.84, 2.19)	1.64 (0.84, 3.22)	1.33 (0.58, 3.03)	1.26 (0.43, 3.66)	1.05 (0.55, 2.03)	0.54 (0.25, 1.18)	0.99 (0.64, 1.55)
**>36.2% (n=524)**	0.78 (0.42, 1.42)	1.48 (0.66, 3.35)	0.41 (0.14, 1.22)	0.73 (0.22, 2.46)	0.77 (0.36, 1.65)	0.91 (0.42, 1.94)	0.97 (0.60, 1.57)
**p-value for linear trend**	0.96	0.07	0.37	0.74	0.74	0.43	0.77
**p-value for omega-3**	***0.041****	0.60	***0.007*****	0.14	0.73	***0.01****	***0.047****

CVD, cardiovascular disease; CHD, coronary heart disease; CI, confidence interval.

*P<0.05; **P<0.01

All significant hazard ratios/P-values are shown in bold italics.

A. Unadjusted model, B. Adjusted for all variables in Table 1 except history of CVD, C. Adjusted for omega-3 index and all variables in Table 1 except history of CVD
